# Using item response theory to investigate the structure of anticipated affect: do self-reports about future affective reactions conform to typical or maximal models?

**DOI:** 10.3389/fpsyg.2015.01438

**Published:** 2015-09-24

**Authors:** Leonidas A. Zampetakis, Manolis Lerakis, Konstantinos Kafetsios, Vassilis Moustakis

**Affiliations:** ^1^Management Systems Laboratory, School of Production Engineering and Management, Technical University of CreteChania, Greece; ^2^The Applied Psychology Laboratory, Department of Psychology, University of CreteRethymnon, Greece

**Keywords:** anticipated affect, unfolding, item response theory, ideal point models, dominance models

## Abstract

In the present research, we used item response theory (IRT) to examine whether effective predictions (anticipated affect) conforms to a typical (i.e., what people usually do) or a maximal behavior process (i.e., what people can do). The former, correspond to non-monotonic ideal point IRT models, whereas the latter correspond to monotonic dominance IRT models. A convenience, cross-sectional student sample (*N* = 1624) was used. Participants were asked to report on anticipated positive and negative affect around a hypothetical event (emotions surrounding the start of a new business). We carried out analysis comparing graded response model (GRM), a dominance IRT model, against generalized graded unfolding model, an unfolding IRT model. We found that the GRM provided a better fit to the data. Findings suggest that the self-report responses to anticipated affect conform to dominance response process (i.e., maximal behavior). The paper also discusses implications for a growing literature on anticipated affect.

## Introduction

Anticipated affect (AAF), people’s predictions of their affective reactions to future events (Loewenstein and Lerner, 2003) is considered a central component of human decision-making, as it enhances individuals’ flexibility in novel situations while it provides a motivational base for self-regulation and action (Mellers and McGraw, 2001; Wilson and Gilbert, 2003). Despite the importance of AAF in human decision-making and wellbeing, and the amount of attention that scholars have devoted, there exists relative little research that empirically tackles the psychological process of responding to self-reported items used in AAF studies. Understanding the process that underlies item responses may provide a better insight into the nature of AAF itself and emotion self-reports in general (Robinson and Clore, 2002).

Item response theory (IRT) models continue to reshape how responses to psychological measures are understood. This also holds for the procedure followed in responding to emotion questions. Specifically, IRT models assume that respondents and items (emotion ratings in this case) can be jointly represented as locations on a latent unidimensional continuum. Recent research suggests there is a correspondence between patterns of responding in self-report scales and the type of underlying behavior that the scale purports to measure. Drawing on the work of Coombs (1964) and Davison (1977), Tay et al. (2009) have proposed a theoretical framework regarding the conceptual correspondence between maximal behaviors (i.e., what an individual can do) and typical behaviors (i.e., what an individual commonly engages in) and IRT models applied to measuring those constructs. The results of that research has shown that assessment of maximal behavior is better described by dominance item response process (i.e., monotonic) whereas self-reported typical behavior is better modeled as an ideal point or and unfolding item response process (i.e., non-monotonic).

In the same vein, Drasgow et al. (2010) argued that in responding to self-report items, introspection takes place so that respondents tend to endorse items that are better self-descriptors of their typical characteristics. In such a situation, the response process follows a matching algorithm of responding that is, a non-monotonic response process (Tay and Drasgow, 2012b). Thus, dominance response models that describe self-report patterns may not be accurately describing processes involving introspection. Such models are most sensibly applied when the aim is to determine the threshold or capacity limits of individual attributes like cognitive or physical ability (Stark et al., 2006).

**FIGURE 1:**
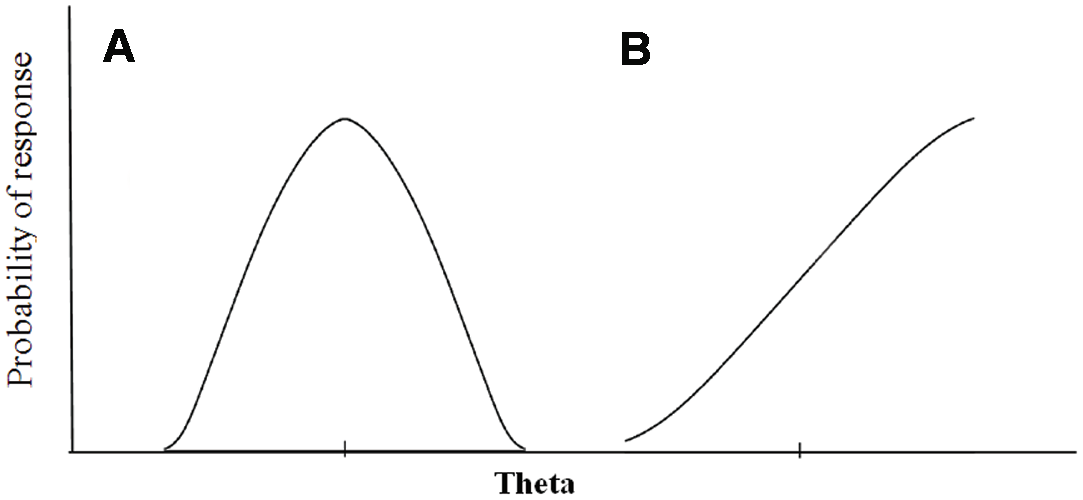
**Graphical representation of the unfolding versus dominance response process: **(A)** item response function for an ideal point response process and **(B)** item response function for a dominance response process**.

In **Figure 1**, we graphically represent the patterns behind the two different response processes. A single-peaked, non-monotonic function is the key feature that distinguishes unfolding IRT models (**Figure 1A**) from traditional, dominance or cumulative IRT models (**Figure 1B**; Stark et al., 2006).

In the case of affective reports, Robinson and Clore (2002) consider two major retrieval processes that contribute to emotion self-reporting. The first relates to episodic emotional knowledge. Episodic knowledge allows for a person to be consciously aware of an earlier experience in a certain situation at a certain time (Tulving, 1993). In the case of on-line affect (“how I am feeling now”) affect is accessible, and individuals tend to use experiential information and introspection in order to endorse items (emotion words for instance) that are closer descriptors of their own states. Therefore, reflecting typical behavior, a non-monotonic ideal point response process is expected to characterize responses to episodic emotion self-reports. Research has provided support for this idea (Tay and Drasgow, 2012b).

The second retrieval processes relates to semantic emotion knowledge. In cases where episodic emotion knowledge is difficult or impossible to retrieve as in the case of prospective emotion reports, individuals are expected to base their responses on semantic emotion knowledge (i.e., general beliefs of how situations are likely to influence emotion). Semantic emotional knowledge is general in nature and does not depend on time or on place (Tulving, 1993). When individuals respond to prospective or hypothetical emotion reports, experience is not accessible to introspection.

Putting the foregoing evidence together leads to following line of reasoning: AAF of a distant future event may relate more to semantic knowledge and include less detail related to specific properties. It is, therefore, plausible, this kind of emotion reports may create expectations as to whether a “right” response is demanded (i.e., a maximal behavior). In cases such as this, then, a monotonic, dominance response process applies, where individuals with high latent trait levels “dominate” items and are likely to answer affirmatively. Nevertheless, this idea has not been tested empirically. Moreover, if our reasoning proves correct, it suggests that anticipated positive affect (PA) and negative affect (NA) are independent constructs, extending the evaluative space model (ESM) of online affect (Cacioppo and Berntson, 1994; Larsen and McGraw, 2011). The ESM proposes two independent positivity and negativity dimensions and that pleasantness and unpleasantness are not necessarily reciprocal, or mutually exclusive.

Understanding the process that underlies item response in AAF is important, for both related psychological theory and measurement (Tay et al., 2009). An important theoretical question for advancing research in this area is whether the response process by which answering to AAF items conforms to dominance or ideal-point response models. Therefore, in order to address this substantial theoretical question, we undertook an empirical analysis where a dominance IRT model was evaluated against an unfolding IRT model using the largest sample of respondents in the context of AAF.

The aim of the present study was to provide evidence concerning the pattern of response that people follow when responding to hypothetical emotion reports. It is important to note, however, that we do not intend to examine the accuracy of people’s affective forecasts (i.e., our study does not concern actual emotional experiences which are compared to predicted emotional experiences). We used the responses of a large number of participants (*N* = 1624) who were asked to report their AAF in a hypothetical situation that has clear implications for emotion experienced in the future, namely, the process of starting their own business.

## Materials and Methods

### Participants and Procedure

The present study was part of a larger project investigating the role of anticipated emotions in student business start-up in Greece. Survey data were collected from 1624 students from six Greek universities. Surveys were administrated to participants through personal contact by the study authors. A variety of recruitment methods were used, including word of mouth, advertising through social network sites, and course credit. The study was described as examining “Factors affecting students’ career choice”. Participants were informed that anonymity was guaranteed and that they had the option to withdraw from the study at any moment. Participants provided their informed consent, prior filling the survey. The survey used was approved by the universities’ Human Research Ethics Committee.

The survey instrument contained items representing the theoretical constructs along with demographic data. Items referring to the same construct were positioned in different locations throughout the questionnaire. Data collection took place at the end of the 2012 spring semester. There were no missing values for the focal variables of interest (i.e., PA and NA).

The sample consisted of 764 male students (47%) with a mean sample age of 21.09 years (*SD* = 2.37). Ninety-eight participants (6%) were postgraduate students. The majority (36.7%) were engineering students followed by social science students (e.g., psychology and education; 25%), business students (22.8%), and science students (e.g., chemistry, physics, and medicine; 15.5%). Thirty-eight percent of the participants reported that one of their parents owned full time business most of the time, while they were growing up, 78% reported that they know an entrepreneur in their close environment.

### Measurement

#### Anticipated PA and NA

To assess participants’ AAF we used 20 items (10 positive and 10 negative) from the Positive and Negative Affect Schedule (PANAS; Watson et al., 1999). The instructions were as follows: “This is an effort to combine research into factors affecting students’ career choice. Your participation is not obligatory; you will answer a questionnaire without filling in anything that will identify you, or your department. Please imagine a situation where you are involved in on-going but not yet operational business start-up. You have invested no money, no income has been made and the firm is not a legal entity. To what extent do you anticipate feeling this [emotion term] from the aforementioned hypothetical situation?” Responses were made on a 5-point scale (1-very slightly or not at all to 5-extremely).

The 10 PA items were: interested, excited, strong, enthusiastic, proud, alert, inspired, determined, attentive, and active. Cronbach’s reliability coefficient for PA was 0.87. The 10 NA items were: distressed, upset, guilt, scared, hostile, irritable, ashamed, nervous, jittery, and afraid. Cronbach’s reliability coefficient for NA was 0.80.

In order to estimate the graded response model (GRM), we reversed the scoring for the 10 NA items so that high scores equal low NA. This has been applied in previous research (Brown and Marshall, 2001). In our case, Cronbach’s reliability coefficient for the 20 items (including the reverse scored items) was 0.84.

### Assessing Dimensionality

All IRT models assume that the latent trait construct space is either strictly unidimensional, or as a practical matter, dominated by a general underlying factor. The assumption of unidimensionality means that only a single latent trait sufficiently predicts individuals’ test performance, or that only one construct is being measured and only one construct explains individual performance.

In practice, however, this assumption does not hold in a strict sense; the unidimensionality assumption is considered to be satisfied when a single primary latent variable accounts for test performance. Drasgow and Hulin (1990) suggested that the unidimensionality assumption is reasonably met if there is a dominant factor in the data, that is, IRT models will perform well as long as the latent variable being measured is dominant over others. As a rule of thumb, the first factor has to account for at least 20% of the total variance for the item parameters to be stable (Reckase, 1979). This rule has been applied by many researchers with different multidimensional constructs (Cooper and Petrides, 2010; Zampetakis, 2011 – for emotional intelligence; or Miguel et al., 2013 – for Career decision scale).

We examined the amount of variance explained by the first factor using exploratory factor analysis (EFA) and principal axis factoring. The number of factors extracted was based on the results of the minimum average partial test method (MAP; O’Connor, 2000) as implemented in the FACTOR software (v. 10.3; Lorenzo-Seva and Ferrando, 2006). Specifically, in our deployment of FACTOR software we used an ordinal treatment of items and the polychoric correlation matrix. Polychoric correlations rest on the assumption that the observed categories function as proxies for bivariate normal continuous phenomena.

### IRT Models and Software

Item parameters along with item and test information functions for the generalized graded unfolding model (GGUM) were estimated with the GGUM2004 (version 1.1) computer program using a marginal maximum likelihood (MML) approach (Roberts et al., 2000). Item parameters for the GRM were estimated with the MULTILOG (version 7.0.3) computer program (Thissen, 2003) using a MML approach and a maximum a posteriori algorithm for person-parameter estimation. We used software defaults for the GGUM2004 and MULTILOG. As previously stated, the application of dominance IRT models requires reverse scoring, where this is not the case for unfolding models. Following established analytic procedures (Tay et al., 2011) negatively valenced items were reverse coded first.

Following Chernyshenko et al. (2001), model-data fit for both GGUM and GRM were examined using fit plots and chi-square goodness of fit tests for single items, pairs, and triplets adjusted (to a sample size of 3000) to χ^2^/df fit statistic. Chernyshenko et al. (2001) demonstrated the usefulness of fit plots by showing that some kinds of misfit can be detected by fit plots but not by summary statistics. Drasgow et al. (1995) have shown that good fitting models have adjusted chi-square to degree of freedom ratios of less than 3 for singlets, doublets, and triplets. The MODFIT (version 2.0) computer program (Stark, 2007) was used to compute chi-square statistics and fit plots.

We compared the relative fit of the ideal point and dominance models with the adjusted χ^2^/df (Tay et al., 2011).

## Results

### Descriptive Statistics and Dimensionality

**Table 1** presents means, standard deviations, and Pearson correlations of responses to the 20 PA and NA adjusted PANAS indicators. The correlation between PA and NA was weak although statistically significant (*r* = -0.21, *p* < 0.001) and in line with the results reported in previous studies (e.g., Watson et al., 1999 have reported low to moderate correlations between the PA and NA scales for online affect, ranging from *r* = -0.12 to -0.23). Furthermore, the PA items correlate with each other only at modest levels (*r*_mean_ = 0.41, *r*_min_ = 0.23, and *r*_max_ = 0.57). This is also the case of the NA (*r*_mean_ = 0.28, *r*_min_ = 0.07, and *r*_max_ = 0.58). Students anticipated feeling more PA (*M* = 3.92, *SD* = 0.79) compared to NA (*M* = 2.26, *SD* = 0.72) [*F*(1,1623) = 3287.68, *p* < 0.001].

**Table 1:** Means, standard deviations, and product moment correlations.

		*M*	*SD*	1	2	3	4	5	6	7	8	9	10	11	12	13	14	15	16	17	18	19	20
1	Interested	4.27	1.11	–																			
2	Excited	3.52	1.23	**0.42^∗∗^**	–																		
3	Strong	3.76	1.12	**0.38^∗∗^**	**0.29^∗∗^**	–																	
4	Enthusiastic	3.89	1.22	**0.57^∗∗^**	**0.46^∗∗^**	**0.39^∗∗^**	–																
5	Proud	3.73	1.25	**0.41^∗∗^**	**0.32^∗∗^**	**0.43^∗∗^**	**0.55^∗∗^**	–															
6	Alert	4.13	1.16	**0.38^∗∗^**	**0.23^∗∗^**	**0.35^∗∗^**	**0.31^∗∗^**	**0.34^∗∗^**	–														
7	Inspired	3.61	1.19	**0.42^∗∗^**	**0.36^∗∗^**	**0.37^∗∗^**	**0.49^∗∗^**	**0.44^∗∗^**	**0.36^∗∗^**	–													
8	Active	3.96	1.18	**0.41^∗∗^**	**0.33^∗∗^**	**0.43^∗∗^**	**0.43^∗∗^**	**0.42^∗∗^**	**0.41^∗∗^**	**0.45^∗∗^**	–												
9	Determined	4.20	1.04	**0.46^∗∗^**	**0.31^∗∗^**	**0.47^∗∗^**	**0.45^∗∗^**	**0.42^∗∗^**	**0.41^∗∗^**	**0.44^∗∗^**	**0.52^∗∗^**	–											
10	Attentive	4.17	1.09	**0.48^∗∗^**	**0.32^∗∗^**	**0.37^∗∗^**	**0.40^∗∗^**	**0.38^∗∗^**	**0.44^∗∗^**	**0.40^∗∗^**	**0.52^∗∗^**	**0.56^∗∗^**	–										
11.	Distressed	1.88	1.10	-0.24^∗∗^	-0.13**^∗∗^**	-0.24**^∗∗^**	-0.28**^∗∗^**	-0.22**^∗∗^**	-0.19**^∗∗^**	-0.19**^∗∗^**	-0.25**^∗∗^**	-0.17**^∗∗^**	-0.18**^∗∗^**	–									
12	Upset	3.07	1.22	-0.09	0.08	-0.03	0.06	0.06	-0.11	-0.10	0.03	0.03	-0.09**^∗∗^**	**0.21^∗∗^**	**–**								
13	Guilt	1.71	1.12	-0.22^∗∗^	-0.11**^∗∗^**	-0.21**^∗∗^**	-0.24**^∗∗^**	-0.24	-0.20**^∗∗^**	-0.16**^∗∗^**	-0.15**^∗∗^**	-0.20**^∗∗^**	-0.18**^∗∗^**	**0.33^∗∗^**	**0.13^∗∗^**	–							
14	Scared	2.38	1.24	-0.07^∗∗^	0.05**^∗^**	-0.18**^∗∗^**	-0.07**^∗∗^**	-0.09**^∗∗^**	-0.02	-0.05**^∗^**	-0.09**^∗∗^**	-0.16**^∗∗^**	-0.05**^∗^**	**0.24^∗∗^**	**0.36^∗∗^**	**0.32^∗∗^**	–						
15	Hostile	1.73	1.10	-0.21^∗∗^	-0.05**^∗^**	-0.19**^∗∗^**	-0.12**^∗∗^**	-0.12**^∗∗^**	-0.21**^∗∗^**	-0.12**^∗∗^**	-0.09**^∗∗^**	-0.14**^∗∗^**	-0.18**^∗∗^**	**0.22^∗∗^**	**0.13^∗∗^**	**0.36^∗∗^**	**0.27^∗∗^**	–					
16	Irritable	2.35	1.29	-0.05	0.03	-0.05**^∗^**	-0.03	0.01	-0.08**^∗∗^**	-0.05**^∗^**	-0.09**^∗∗^**	-0.05**^∗^**	-0.07**^∗^**	**0.20^∗∗^**	**0.31^∗∗^**	**0.25^∗∗^**	**0.21^∗∗^**	**0.41^∗∗^**	–				
17	Shame	1.60	1.11	-0.23^∗∗^	-0.07**^∗∗^**	-0.20**^∗∗^**	-0.21**^∗∗^**	-0.21**^∗∗^**	-0.32**^∗∗^**	-0.19**^∗∗^**	-0.20**^∗∗^**	-0.22**^∗∗^**	-0.28**^∗∗^**	**0.28^∗∗^**	**0.07^∗∗^**	**0.43^∗∗^**	**0.19^∗∗^**	**0.35^∗∗^**	**0.31^∗∗^**	–			
18	Nervous	2.99	1.29	0.08	0.07	-0.05	0.08	0.11	0.06**^∗^**	0.07**^∗^**	-0.01	0.01	0.01	**0.09^∗∗^**	**0.37^∗∗^**	**0.08^∗∗^**	**0.33^∗∗^**	**0.18^∗∗^**	**0.42^∗∗^**	**0.15^∗∗^**	–		
19	Jittery	2.33	1.31	-0.07^∗∗^	0.05**^∗^**	-0.19**^∗∗^**	-0.08**^∗∗^**	-0.05**^∗^**	-0.09**^∗∗^**	-0.04	-0.15**^∗∗^**	-0.16**^∗∗^**	-0.11**^∗∗^**	**0.22^∗∗^**	**0.37^∗∗^**	**0.23^∗∗^**	**0.49^∗∗^**	**0.28^∗∗^**	**0.39^∗∗^**	**0.30^∗∗^**	**0.42^∗∗^**	**–**	
20	Afraid	2.53	1.23	-0.01	0.08**^∗∗^**	-0.18**^∗∗^**	-0.04	-0.01	-0.05**^∗^**	0.02	-0.06**^∗^**	-0.12**^∗∗^**	-0.03	**0.20^∗∗^**	**0.32^∗∗^**	**0.21^∗∗^**	**0.48^∗∗^**	**0.17^∗∗^**	**0.24^∗∗^**	**0.22^∗∗^**	**0.38^∗∗^**	**0.58^∗∗^**	–


*N = 1624; positive affect (PA) and negative affect (NA) inter-item correlations are bolded.*

*^∗^Correlation is significant at the 0.05 level (2-tailed).*

*^∗∗^Correlation is significant at the 0.01 level (2-tailed).*

In the current study, the correlation between anticipated PA and NA, was low. However, the correlation coefficient is not considered an appropriate index to test whether PA and NA are separable constructs (Russell and Carroll, 1999). We used a different index; the gradual threshold model (GTM) formula suggested by Priester and Petty (1996). According to the GTM, ambivalence increases in a negatively accelerating manner as the number of conflicting reactions (whichever of the positive or negative reactions are fewer in number) increases. In our case the GTM index had mean value of 6.4 (*SD* = 1.18; minimum = 3.52; maximum = 9.55), providing evidence that the two constructs are independent. Students anticipate feeling both PA and NA from the hypothetical situation of their own business start-up.

We used EFA to determine the suitability of implementing unidimensional IRT models. The Kaiser–Meyer–Olkin (KMO) measure of sampling adequacy was 0.88, indicating that EFA was appropriate for the 20 item PANAS scale and for this sample. With MAP, no further components needed to be extracted from the matrix after the first 2 principal components were removed from the original correlation matrix (Component 1: average partial correlation = 0.0482; Component 2: average partial correlation = 0.0194; and Component 3: average partial correlation = 0.0293). This finding indicated that the variance of the items due to the third common factor was smaller than the variance due to random factors, suggesting that only two factors should be extracted from the original data.

The first three eigenvalues were 5.379, 3.294, and 1.266, accounting for 26.89, 16.47, and 6.31 of the total variance, respectively. Thus, the first factor accounted for at least 20% of the total variance and the unidimensionality assumption is reasonably met (Drasgow and Hulin, 1990; Cooper and Petrides, 2010; Zampetakis, 2011).

### Model Data Fit

To determine which model better fit the data, we examined graphical fit plots and statistical tests of goodness of fit. In **Table 2**, we present the adjusted (*N* = 3000) χ^2^/df fit statistics provided by MODFIT.

**Table 2:** Frequencies of the values of the adjusted (*N* = 3000) chi-square statistic to degrees of freedom from the model fit analysis.

Model	<1	1 to <2	2 to <3	3 to <4	4 to <5	5 to <7	>7	Mean	*SD*
**Graded response model (GRM)**									
Singlets	20	0	0	0	0	0	0	0.02	0.11
Doublets	1	12	22	20	43	46	46	7.02	7.97
Triplets	0	14	98	236	236	283	273	5.89	3.22
**Generalized graded unfolding model (GGUM)**									
Singlets	10	0	2	0	1	4	3	3.03	3.49
Doublets	0	1	10	20	29	42	88	7.61	4.25
Triplets	0	0	13	80	176	456	415	6.58	2.15


**FIGURE 2:**
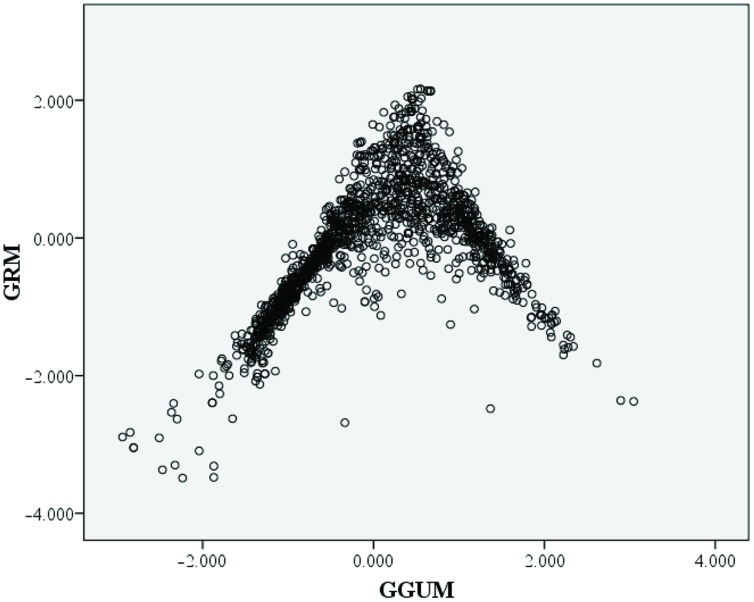
Scatter-plot comparisons of the theta value estimates from graded response model (GRM) and generalized graded unfolding model (GGUM).

Results suggest that the GRM produces a better model-data fit compared to the GGUM. Although the absolute fit of the two models is above to the recommended χ^2^/df fit criteria it is suggested that when moderate sample sizes are used (i.e., 1000–1500), a fixed cut-off value is insufficient to ascertain IRT model-data because of high Type I error rates (Tay and Drasgow, 2012a). The theta estimates for the GRM and GGUM showed some correlation (*r* = 0.47). Examining the scatterplot matrix of the individual estimates (**Figure 2**) depicted an inverted funnel distribution. The incongruence between the GRM and GGUM occurred at higher levels of theta.

**FIGURE 3:**
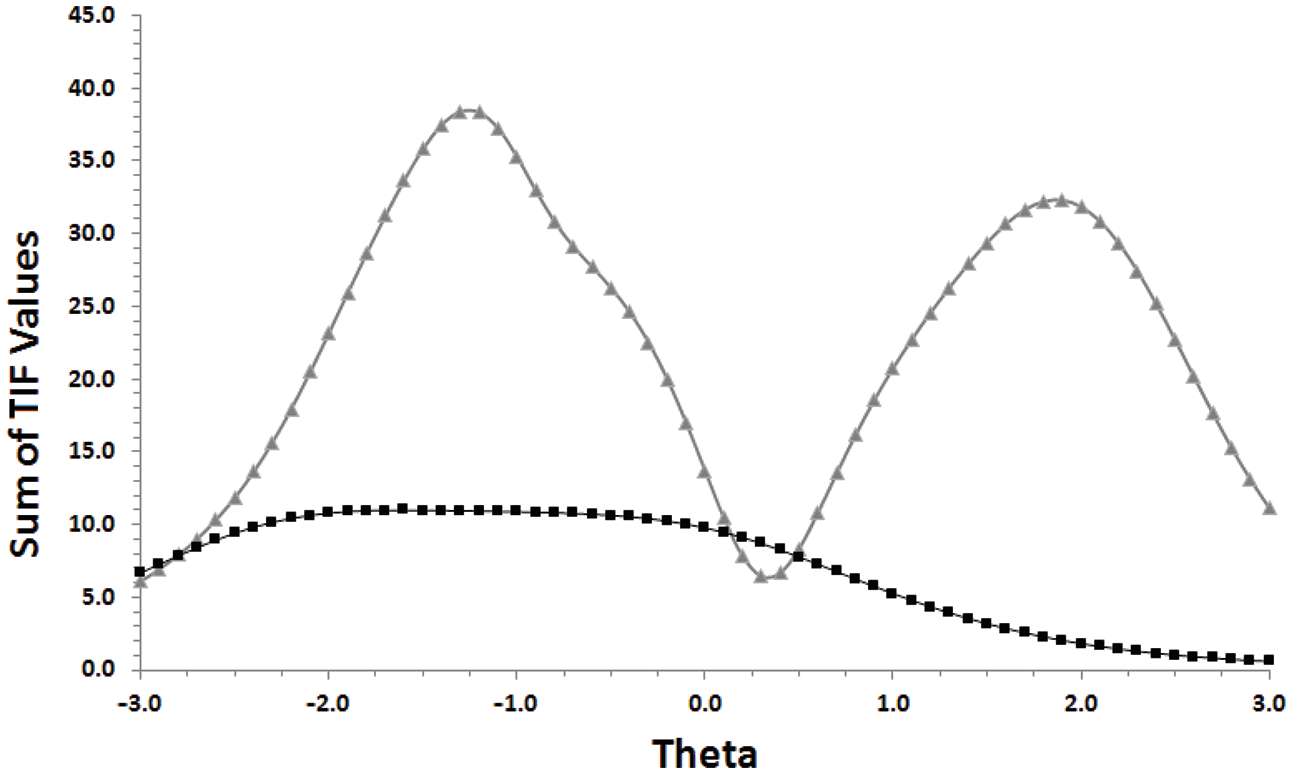
**Overlay of test information function (TIF) plots for the anticipated Positive and Negative Affect Schedule (PANAS) scale in the GGUM and GRM.** The lightest-colored line is the TIF for the GGUM.

An examination of the test information functions (**Figure 3**) which provide an indication of measurement precision across levels of theta, showed that precision is best for the GRM only at low levels of theta whereas the GGUM showed precision at distinctly low and high levels of theta.

## Discussion

The aim of the present study was to examine the response process that people follow when responding to hypothetical emotion reports during affective forecasting. The results were in line with our expectations that a dominance IRT model, namely the GRM (Samejima, 1969), would fit the response process for AAF in a hypothetical emotion situation better than the GGUM (Roberts et al., 2000). We take this as evidence to suggest that when people report their AAF, their judgment is influenced by normative beliefs about what is considered an appropriate emotional response. Under these situations a benchmark is established due to the fact that there is a common consensus to what is the most appropriate, or correct, response. Thus, the process that underlies item responses is a dominant one.

Tay and Drasgow (2012a) have established that an ideal point process is relevant for the description of response to online affect. Our results complement this line of research suggesting that in the case of AAF the response process that people follow is based on dominance models. Furthermore, our results may provide some insights into the relationship between AAF and self-regulatory outcomes (Mellers and McGraw, 2001). Specifically, our results suggest that AAF is a cognitive representation of a future emotional state which is largely influenced by normative beliefs about what is considered an appropriate emotional response. These beliefs relate to what may be socially appropriate but do not necessarily depend on the anticipated reaction of others; the issue here is “what should I feel?”, rather “what do others think of my prediction?” Researchers should take this information into account when performing manipulation checks and interpreting results of studies on AAF.

Results suggest that the valence of AAF is best conceptualized as two separate dimensions. Stated differently, we provide evidence that the structure of AAF is bivariate: anticipated positivity and negativity are separable. Our results are in line with the ESM which proposes two independent positivity and negativity dimensions (Cacioppo and Berntson, 1994; Larsen and McGraw, 2011). Therefore, and extending the ESM model to affective prediction, three anticipated affective states have to be differentiated: positive, negative, and emotionally ambivalent (i.e., positive and negative).

Every circle represents a person’s trait estimates under the two perspectives.

Although this study shines new light on the structure of AAF, it has several limitations.

First, in the present research we used an IRT perspective for the comparison of measurement models, which is an indirect approximation of the item response process with several limitations. For example, in IRT models the distribution of the latent trait is often assumed to be normally distributed but this may not be true for many psychological scales. Moreover, the relative fit of the models do not describe if any single individual utilizes such a response process.

Second, our sample represents a cross-sectional survey of individuals and our current investigation is limited to the relation between AAF to a hypothetical situation involving business start-up of Greek students and response patterns, as assessed through self-reports. Our results and implications should be restricted to that topic. An important avenue for future research and review efforts would consist of examining whether our conclusions also hold for other hypothetical situations and for different cultures.

Third, we cannot completely rule out the possibility that the polytomous IRT model may mask an ideal point response process among the PANAS item options. More specifically, although by design, the PANAS model is based on the premise that the dimensions of PA and NA are independent (Watson et al., 1999) it is also founded on the assumption of a basic bipolar valence dimension containing the negatively correlated endpoints of happiness and sadness. According to the bipolar perspective proposed by Russell and Carroll (1999) and others, emotional experiences can be either positive or negative. Because the emotion state of an individual at any given point in time is located at a single point in the affect space the subjective sense of positivity and negativity is posited to be mutually exclusive. In the current study, in order to estimate the GRM we reversed the scoring for the 10 NA items the reliability coefficient for the 20 items (including the reverse scored items) was high providing evidence that they describe the same quantities along a single valence dimension (i.e., they form a single cumulative scale) suggesting that anticipated NA is the opposite of anticipated PA. Furthermore, the pattern of correlations presented in **Table 2** suggests that anticipated PA and NA form a single bipolar factor, albeit a weak 1; PA and NA emotion words correlate with each other only at modest levels, and correlate with emotions of the opposite valence to this same degree (Diener et al., 1995). This suggests that less positive valence implies more negative valence, which leaves no room for mixtures. Future research could address this issue.

Fourth, with regard to the measures, all items have five response options, including the middle category, neither agree nor disagree. According to researchers including Andrich and Styles (1998) and Tay et al. (2009) the middle category does not necessarily function as the mid-point between two adjacent response options. Future research concerning the structure of AAF could find meaningful ways to collapse responses instead using all five response options. Moreover, other IRT models could have been selected, especially for this kind of data. Non-parametric cumulative IRT models were not used in this study, although they provide a broad-spectrum and flexible data analytic framework and their use in the field of cognitive and non-cognitive measurement is well accepted (Meijer and Baneke, 2004). Future research could also address this.

## Conclusion

Tay et al. (2009) have proposed a theoretical framework suggesting that measurement scales in psychology should be scored based on the response models that fit the data best. We provide evidence that when people report their AAF, the process that underlies item responses is a dominant one. This suggests that emotions terms are not located on a unidimensional construct with intense happiness and sadness located at the endpoles. Rather the structure of AAF is bivariate: anticipated positivity and negativity are separable dimensions. Conceptually, this allows one to account for complex anticipated emotional states such as mixed anticipated happiness and sadness.

## Conflict of Interest Statement

The authors declare that the research was conducted in the absence of any commercial or financial relationships that could be construed as a potential conflict of interest.
